# Integrated Metagenomic and Metabolomic Profiling Identifies Predictive Biomarkers for Overweight Status in a Mongolian Population

**DOI:** 10.3390/microorganisms14050946

**Published:** 2026-04-22

**Authors:** Zhixin Zhao, Xiaoyan Wang, Fang Wen, Feiyan Zhao, Mengdi Zhang, Bilige Menghe

**Affiliations:** 1 Inner Mongolia Key Laboratory of Dairy Biotechnology and Engineering, Inner Mongolia Agricultural University, Hohhot 010018, China; nmzzx13644875037@163.com (Z.Z.); wxy15128975787@163.com (X.W.); 13190518403@163.com (F.W.); 15754881670@163.com (F.Z.); zhangmengdi1108@163.com (M.Z.); 2 Key Laboratory of Dairy Products Processing, Ministry of Agriculture and Rural Affairs, Inner Mongolia Agricultural University, Hohhot 010018, China; 3 Key Laboratory of Dairy Biotechnology and Engineering, Ministry of Education, Inner Mongolia Agricultural University, Hohhot 010018, China; 4 College of Pharmacy, Inner Mongolia Medical University, Hohhot 010110, China

**Keywords:** overweight Mongolians, gut microbiota, serum metabolites, potential targeted biomarkers

## Abstract

Mongolians have high overweight prevalence linked to their nomadic lifestyle and diet, but gut microbiota studies in this population are scarce. This study used fecal metagenomic and serum metabolomic analyses of 96 Mongolian participants (normal-weight n = 55, overweight n = 41) to characterize gut microbiome alterations and identify weight-related biomarkers. The analyses revealed that *Parabacteroides distasonis*, *Barnesiella intestinihominis*, and *Alistipes onderdonkii* were significantly reduced in overweight individuals (*p* < 0.05). Concurrently, the metabolites such as beta-cryptoxanthin, p-cresol, and ribothymidine were significantly down-regulated in the overweight group (*p* < 0.05). Random forest models from the three datasets showed a strong diagnostic ability for microbial families (AUC > 0.70). A subsequent integrated multi-kingdom classifier that combined microbiota and metabolite data achieved the highest performance (AUC = 0.818). Key features with high predictive contributions were identified, including *Lactobacillus crispatus*, *Alistipes onderdonkii*, and *Parabacteroides distasonis*, and metabolites, such as beta-cryptoxanthin, p-cresol, and picolinic acid. These results show the random forest model has high predictive value for distinguishing normal weight and overweight individuals. In summary, this study identified specific gut microbiota and serum metabolomic profiles linked to overweight in Mongolians. Multi-omics integration established a diagnostic biomarker model, laying a theoretical basis for microbiome-targeted weight management interventions.

## 1. Introduction

The Mongolian people are characterized by a distinct lifestyle, with the majority of the population inhabiting rural areas and practicing pastoralism for their livelihood. Their dietary habits are characterized by a preference for high-protein and high-fat foods. This animal-based dietary pattern is consistent with nomadic lifestyles. Therefore, meat, milk, and their products are the primary sources of caloric intake for this population. The composition and diversity of the human gut microbiota are influenced by a multitude of factors, including dietary habits [1], lifestyle [2], genetic background [3], region [4], and ethnicity [5,6]. Among these, diet is recognized as a particularly dominant factor [7]. Given the dietary patterns of the Mongolian population, their high-calorie intake is not only a significant contributor to the prevalence of overweight individuals but is also hypothesized to induce corresponding shifts in the gut microbiota [7,8].

Gut dysbiosis is increasingly recognized as a key factor contributing to weight gain. Studies have demonstrated significant differences in the composition and diversity of gut microbial communities between overweight and normal-weight individuals, accompanied by corresponding alterations in host metabolism [9,10]. Individuals whose weight exceeds the normal range typically exhibit reduced gut microbial diversity compared with their normal-weight counterparts [11]. Excess body mass is frequently associated with depletion of gut *Bifidobacterium* [12], and lower fecal levels of this genus have been observed in children with progressive weight gain [13]. Liu et al. [14] reported that microbial genes associated with “glutamate/glutamine metabolism” and “branched-chain amino acid transport” were significantly reduced in the obesity group, while serum levels of glutamate and both branched-chain and aromatic amino acids were concurrently elevated, exhibiting a significant negative correlation with the abundance of *Bacteroides thetaiotaomicron*. Animal models corroborate these human studies, demonstrating that genetically obese mice harbor less diverse gut microbiota than normal mice [15,16]. In a diet-induced obesity mouse model, supplementation with *Roseburia hominis* can significantly reduce body weight and fat content, improve blood glucose control and enhance insulin sensitivity, as well as lower serum triglyceride and cholesterol levels to reduce the risk of cardiovascular diseases [17].

The convergence of recent advances in multi-omics technologies with the imperative to understand health characteristics in distinct ethnic populations underscores the significance of elucidating the gut microbial profiles of overweight Mongolian individuals. Therefore, this study was designed to compare overweight and normal-weight Mongolian cohorts. Employing an integrated dual-omics approach, a random forest prediction model was used to identify potential gut microbiota and serum metabolites associated with intestinal alterations in the overweight group. This study aimed to comprehensively characterize the alterations in the gut microbiome composition and metabolite profiles of overweight Mongolian individuals. This study aimed to identify key microbiological features associated with overweight status and evaluate their diagnostic potential, thereby providing important insights into future intervention strategies.

## 2. Materialsand Methods

### 2.1. Trial Design and Participant Recruitment

The study design was based on established methodologies from similar clinical and microbiome investigations [18,19,20]. A total of 110 participants were recruited, including 55 overweight individuals and 55 normal-weight controls. Sample size was calculated using body weight-related indicators reported in previous studies. With a preset between-group difference of 4 in dietary intake, a standard deviation of 5, a two-sided α = 0.05, and a statistical power of 90%, a minimum of 40 subjects per group was estimated, accounting for a dropout rate of less than 20%. Normal-weight participants were slightly oversampled to ensure sufficient statistical power for subgroup analyses of gut microbiota diversity.

**Figure 1:**
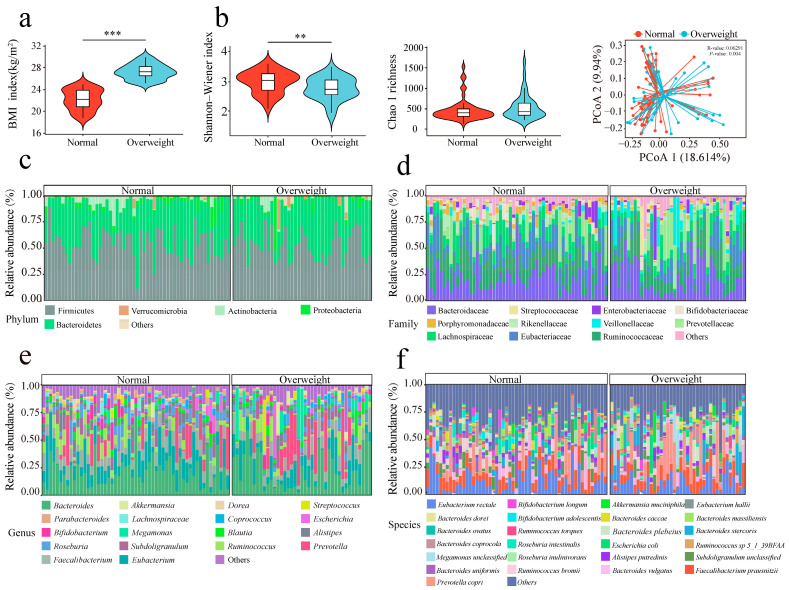
The diversity and composition of the microbial communities in the overweight group compared to the normal group. (**a**) The differences in BMI between the overweight group and the normal group were compared. (**b**) Alpha and beta diversity of the gut microbiota in overweight group (n = 41) and normal group (n = 55) groups. The violin plot represents the distribution state of each group of data and the horizontal line inside the box represents the median. ** *p* < 0.01 and *** *p* < 0.001 (Mann–Whitney-Wilcoxon test). Principal coordinate analysis (Bray–Curtis dissimilarity) illustrates significant separation in microbial community structure between groups; the result of the Adonis test (999 permutations) is shown. (**c**–**f**) It respectively shows the changes in the microbial community structure of fecal samples in the overweight group and the normal group at the phylum, family, genus, and species levels.

110 participants were screened for eligibility. After excluding 14 individuals (9 who did not meet the inclusion criteria and 5 who declined to participate), the final study cohort comprised 96 individuals. This cohort comprised 41 overweight and 55 normal-weight controls. The two groups were well-balanced in terms of key demographic and anthropometric characteristics (Table S1). The mean age of the overweight group was 44 ± 11.38 years, compared with 40.45 ± 12.46 years in the normal-weight group. In the overweight group, with males accounting for 39.02% and females 60.98%, whereas in the control group, with males accounting for 27.27% and females 72.73%, with an age range of 23–64 years and a median age of 41 years. No statistically significant differences were observed between the two groups in terms of age or sex. (*p* > 0.05). These findings indicate that the overweight and normal-weight groups were well-matched at baseline, which minimized the potential confounding effects of demographic variables in subsequent analyses. As expected, the mean BMI of the overweight group (27.34 ± 1.35 kg/m^2^) was significantly higher than that of the normal-weight group (22.18 ± 1.88 kg/m^2^) (*p* < 0.05, Figure 1a).

The inclusion criteria for the overweight group were as follows: participants must be of Mongolian ethnicity, long-term residents of the Inner Mongolia Autonomous Region, aged ≥ 18 years, and have a 25 kg/m^2^ ≤ Body Mass Index (BMI) < 30 kg/m^2^ [21] regardless of sex. Further requirements included no use of antibiotics or probiotic products within the preceding two months (e.g., yogurt or its Mongolian analogs), no cognitive impairment, and the absence of major diseases. Participants who were pregnant or breastfeeding were excluded. The exclusion criteria were a history of substance abuse, drug dependence, or alcohol addiction; the presence of severe gastrointestinal diseases (e.g., inflammatory bowel disease, intestinal obstruction, colorectal or abdominal tumors, and intestinal tuberculosis); and a history of major gastrointestinal surgery (such as gastrectomy and intestinal resection). Individuals adhering to medically prescribed dietary or exercise regimens or those unable or unwilling to comply with the study protocols were also excluded.

Normal-weight control participants were recruited based on a 18.5 kg/m^2^ ≤ BMI < 25 kg/m^2^, with all other inclusion and exclusion criteria matching those of the overweight group participants.

### 2.2. Basic Parameters and Sample Collection

Venous blood samples were collected from all participants. Immediately after collection, the samples were centrifuged at 1300× *g* for 15 min at 4 °C to isolate the serum. The resulting serum was aliquoted into 2 mL enzyme-free sterile Eppendorf (EP) tubes [22]. Fecal samples were collected from all participants using Longseegen Stool Storage Kits (Guangdong Longsee Biomedical Co., Ltd., Guangzhou, China). The participants were provided with concise instructions and sterile collection devices to prevent contamination during sample collection. All serum and fecal samples were immediately labeled, transported to the laboratory, and stored at −80 °C until analysis.

### 2.3. DNA Extraction and Shotgun Metagenomic Sequencing

Genomic DNA was extracted from fecal samples using the QIAGEN QIAamp Fast DNA Stool Mini Kit (Qiagen GmbH, Hilden, Germany) in strict adherence to the manufacturer’s guidelines. The purity and concentration of the extracted DNA were quantified using a Nanodrop spectrophotometer and a Qubit^®^ 2.0 fluorometer (Thermo Fisher Scientific, Waltham, MA, USA) with the Qubit^®^ dsDNA Assay Kit (Life Technologies, Carlsbad, CA, USA) [23]. DNA integrity was verified by 1.0% agarose gel electrophoresis. DNA samples that met the quality control criteria (concentration > 20 ng/μL; an optical density ratio (at 260 to 280 nm) of 1.8–2.0) were selected for library preparation. Paired-end shotgun metagenomic sequencing was performed on an Illumina NovaSeq 6000 platform (Illumina Inc., San Diego, CA, USA) by Novogene Technology Co., Ltd. (Tianjin, China).

### 2.4. Bioinformatics of Metagenomic Data

Metagenomic shotgun sequencing of fecal samples yielded a total of 639.02 Gb of raw paired-end reads (6.66 ± 0.32 Gb per sample; range: 5.89–7.28 Gb). Low-quality sequences and host-contaminated reads were removed from the raw data using the KneadData quality control pipeline (http://huttenhower.sph.harvard.edu/kneaddata, accessed on 19 May 2025), which incorporates Bowtie2 (V.2.4.4) and Trimmomatic. Following this preprocessing, 628.32 Gb of high-quality paired-end reads were retained for downstream analysis (6.54 ± 0.35 Gb per sample; range: 5.03–7.17 Gb).

High-quality reads were subjected to taxonomic and functional annotation using the HUMAnN 3.0 pipeline (https://huttenhower.sph.harvard.edu/humann/, accessed on 19 May 2025). Taxonomic profiling and relative abundance of species were determined using MetaPhlAn 3 with default parameters. For functional analysis, pan-genomic nucleotide sequences were aligned against the ChocoPhlAn 3 database using Bowtie 2 v.2.4.4 with default settings. Unaligned reads were translated into protein sequences and subsequently matched against the UniRef90 protein database using DIAMOND v2.1.21 with default parameters. The abundance of gene families and coverage of metabolic pathways were quantified using the HUMAnN core algorithm with the MetaCyc database (v29.1), and all other steps were performed using the default parameters, unless otherwise stated.

### 2.5. LC-MS Untargeted Metabolomics Analysis

Sample preparation was performed according to previously established protocols [24,25]. Briefly, the serum samples were thawed at 4 °C and subsequently centrifuged at 12,000× *g* for 10 min. The upper fat layer was carefully removed, and the resulting supernatant was combined with 7 volumes of acetonitrile for protein precipitation. Following vigorous mixing, the precipitated macromolecules were removed by a second centrifugation step at 12,000× *g* for 10 min at 4 °C. The clarified supernatants were dried under vacuum using a rotary evaporator for 10 h. The dried residues were reconstituted in 500 μL of 40% aqueous acetonitrile and clarified by filtration through a 0.22 μm microporous membrane (Agilent, Shanghai, China) prior to analysis. To monitor the stability and repeatability of the analytical system, quality control (QC) samples were prepared by pooling equal aliquots from each experimental sample.

Metabolomic profiling was performed using a Q Exactive Orbitrap Mass Spectrometer (Thermo Fisher Scientific, Shanghai, China) coupled with a liquid chromatography system. Chromatographic separation was achieved using an Acquity HSS T3 column (1.8 μm, 2.1 × 100 mm; Waters, Milford, MA, USA) with a mobile phase flow rate of 0.3 mL/min and an injection volume of 2 μL. In the positive ionization mode, the mobile phases consisted of (A) 0.1% formic acid in water and (B) acetonitrile. The elution gradient was as follows: 0.25 min, 98% A, 2% B; 12 min, 2% A, 98% B; 14 min, 2% A, 98% B; 14.10 min, 98% A, 2% B; 17 min, 98% A, 2% B. For negative ionization mode, the mobile phases were (A) 0.1% ammonium hydroxide in water and (B) acetonitrile, with the following gradient: 3 min, 90% A, 10% B; 6 min, 70% A, 30% B; 8 min, 50% A, 50% B; 12 min, 40% A, 60% B; 14 min, 10% A, 90% B; 15 min, 10% A, 90% B; 16 min, 90% A, 10% B. The total chromatographic runtime was 20 min. Mass spectrometric data were acquired using a data-independent acquisition mode over a mass-to-charge (*m*/*z*) range of 70–1050 Da, enabling the collection of precise mass information for both precursor and product ions.

The raw mass spectrometry data were first converted to mzXML format using the MSConvert tool in the ProteoWizard software package (v3.0.8789). Subsequently, peak detection, peak filtering, and peak alignment were performed using the R package XCMS (v3.12.0) with the following key parameters: bw = 2, ppm = 15, peakwidth = c(5, 30), mzwid = 0.015, mzdiff = 0.01, and method = “centWave”. Metabolite identification was performed according to previously reported criteria, including accurate mass-to-charge ratio (*m*/*z*), retention time, and MS/MS fragmentation pattern matching [26,27]. Systematic error correction was then applied using a support vector regression method based on quality control (QC) samples, and metabolic features with a relative standard deviation (RSD) greater than 30% in the QC samples were filtered out to enhance data reliability.

### 2.6. Quality Control of Metabolomics Data

Metabolite identification was conducted by integrating multiple databases, including HMDB, MassBank, LipidMaps, mzCloud, KEGG, and a custom in-house standard database. Identification was achieved by matching parent ion mass-to-charge ratios (*m*/*z*) and MS/MS fragment ions, with a mass deviation threshold set at ppm < 30. The resulting quantitative metabolite matrix was subjected to multivariate statistical analysis. Non-metric Multidimensional Scaling (NMDS) was employed to assess metabolic differences among sample groups, using Bray–Curtis distance to calculate inter-sample similarity. The fit of the NMDS analysis was evaluated using the stress value (stress value < 0.2 was considered a good fit). Based on the group differences revealed by the NMDS analysis, differential metabolites were screened by combining Variable Importance in Projection (VIP) scores, fold change (FC), and Wilcoxon rank-sum test *p*-values (*p* < 0.05).

### 2.7. Random Forest Model Construction

A random forest model was employed to assess the importance of various features and evaluate the model classification performance. The input data for the model comprised the relative abundances of microbial species, metabolic pathways, and serum metabolites. The model constructs multiple decision trees using bootstrap sampling and employs Gini impurity as the criterion for node splitting. The model performance was quantified using the area under the receiver operating characteristic curve (AUC), and the importance of each feature was determined using the Mean Decrease Accuracy metric. The top 20 species, metabolic pathways, and metabolites ranked by feature importance were selected as key discriminatory variables.

### 2.8. Evaluation of Random Forest Model Performance

The dataset was first partitioned into training (70%) and validation (30%) sets using stratified sampling to maintain the class distribution. The robustness of the model was further evaluated using 10-fold stratified cross-validation, which mitigated the biases introduced by arbitrary data splitting. For performance assessment, ROC curves were generated using sklearn.metrics.roc_curve to derive FPR, TPR, and optimal cutoff values across different decision thresholds. To quantify the statistical uncertainty of model generalization, this study conducted 1000 iterations of Bootstrap resampling. From the resulting distribution of AUC values, this study calculated the 95% confidence intervals, thereby providing a stringent statistical basis for evaluating the efficacy of the model.

### 2.9. Statistical Analysis

Microbial species richness, alpha diversity, and metabolic pathway analyses were performed using the Vegan package in R software (Version 4.0.2). Significant differences in these metrics between the overweight and normal-weight groups were assessed using the Wilcoxon rank-sum test. Principal coordinate analysis (PCoA) was employed to visualize overall shifts in the gut microbiota community structure, and the statistical significance of the differences between groups was evaluated using permutational multivariate analysis of variance (PERMANOVA) with 999 permutations. Student’s *t*-test was used for group comparisons, and Spearman’s rank correlation coefficient was calculated to evaluate the associations between differential microbial species, metabolites, and clinical parameters. Correlation networks were visualized using Cytoscape software v3.10.2. A *p*-value < 0.05 was considered statistically significant. The results are expressed as mean ± standard deviation (SD). All data visualizations were generated using R software (V 4.0.3) and refined for publication using Adobe Illustrator 2024 (version 28.7.1).

## 3. Results

### 3.1. The Gut Microbial Community Structure and Taxonomic Composition Are Altered in Overweight Individuals

Using the Chao1 index to assess gut microbial richness and the Shannon-Wiener index to evaluate gut microbial diversity, the results showed no significant difference in microbial richness (Chao1) between the normal-weight and overweight groups (*p* > 0.05). In contrast, the Shannon-Wiener index, a measure of community evenness and richness, was significantly higher in the normal-weight cohort than in the overweight group (*p* < 0.05). Beta diversity analysis, visualized by PCoA and PERMANOVA, demonstrated a significant divergence in the overall gut microbial community structure between the normal-weight and overweight populations (*p* < 0.05 ANOSIM, *R* = 0.063, *p* = 0.004, Figure 1b). Collectively, these findings indicate that overweight individuals exhibit reduced microbial diversity and gut microbial imbalance.

**Figure 2:**
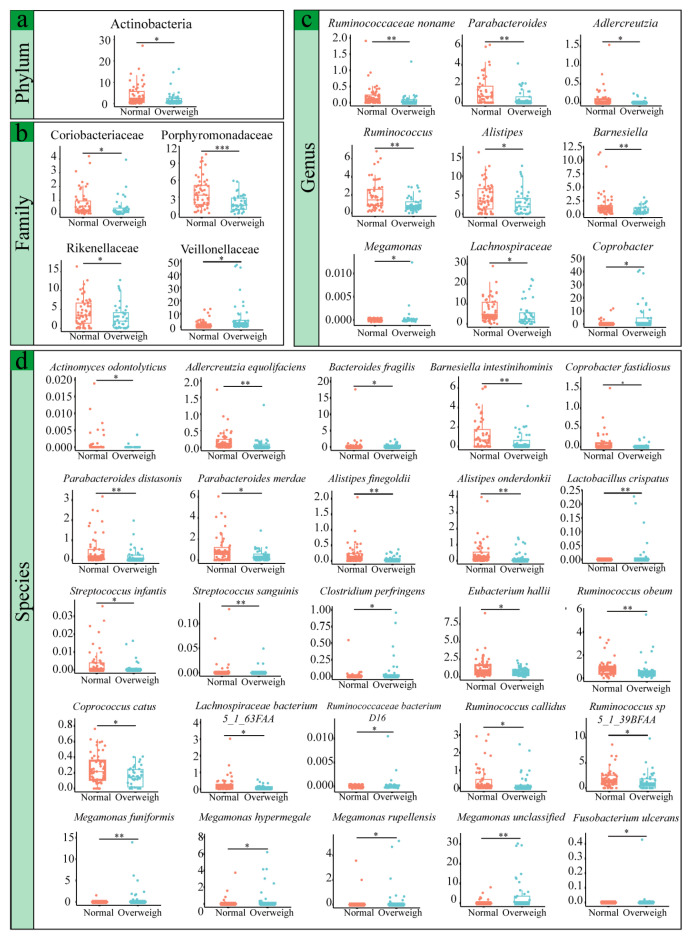
At the levels of phylum, family, genus and species, there are significant differences in the species between the overweight group and the normal group. The Mann–Whitney test was used to statistically evaluate the differences in the species present between the two groups of participants. Corresponding *p*-values indicate data significance. The horizontal line inside the box represents the median, and each dot represents a data point of a participant. (**a**) Phylum level. (**b**) Family level. (**c**) Genus level. (**d**) Species level.* *p* < 0.05, ** *p* < 0.01, *** *p* < 0.001 (Mann–Whitney-Wilcoxon test).

Taxonomic analysis at the phylum level identified five dominant phyla with relative abundances exceeding 1%: Firmicutes, Bacteroidetes, Proteobacteria, Actinobacteria, and Verrucomicrobia (Figure 1c). The relative abundance of Actinobacteria was significantly lower in the overweight group than in the normal-weight group (*p* < 0.05; Figure 2a). There were a total of 11 dominant families with a consensus level and a relative abundance greater than 1% (Figure 1d), including Bacteroidaceae, Ruminococcaceae, Eubacteriaceae, Lachnospiraceae, Prevotellaceae, Veillonellaceae, Rikenellaceae, Porphyromonadaceae, Bifidobacteriaceae, Enterobacteriaceae, and Streptococcaceae. Of these, 4 families showed significant differences between the cohorts. The relative abundances of Coriobacteriaceae, Porphyromonadaceae, and Rikenellaceae were significantly decreased in the overweight population (*p* < 0.05), whereas that of Veillonellaceae was significantly increased (Figure 2b). Further analysis at the genus level identified 18 dominant genera (relative abundance > 1%), nine of which showed significant differences between the groups (Figure 1e). Specifically, the abundances of unclassified genera within *Ruminococcaceae noname*, *Parabacteroides*, *Adlercreutzia*, *Ruminococcus*, *Alistipes*, and *Barnesiella* were significantly lower in the overweight cohort (*p* < 0.05). Conversely, the relative abundance of *Coprobacter* was significantly elevated in overweight individuals (Figure 2c).

At the species level, 430 distinct taxa were identified, with the most dominant species being *Eubacterium rectale* (10.8%), *Faecalibacterium prausnitzii* (9.01%), *Prevotella copri* (8.4%), and *Bacteroides vulgatus* (3.56%) (Figure 1f). Subsequent analysis revealed 25 species that were differentially abundant between the two groups (Figure 2d). The relative abundances of *Barnesiella intestinihominis*, *Parabacteroides distasonis*, *Parabacteroides merdae*, *Alistipes finegoldii*, *Alistipes onderdonkii*, *Streptococcus infantis*, *Eubacterium hallii*, *Ruminococcus obeum*, and *Ruminococcus callidus* were significantly lower in the overweight group than in the normal-weight group (*p* < 0.05; Table S3).

### 3.2. Significant Differences Were Observed in Gut Metabolic Pathways Between Overweight Population and Normal-Weight Population

**Figure 3:**
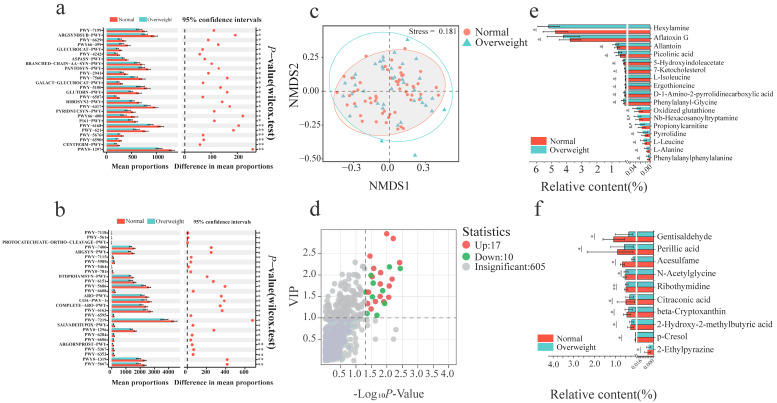
The overweight group and the normal group were compared in gut metabolic pathways and the serum metabolites characteristics. (**a**,**b**) Statistical differences in metabolic pathways were evaluated using the Mann–Whitney test at a confidence interval of 95%. (**c**) Non-metric Multidimensional Scaling score plot (Bray–Curtis distance) showing phage community structures in the two groups, with analysis of similarities (ANOSIM, 999 permutations) and stress value indicated. (**d**) Volcano map showing different metabolites between the overweight group and the normal group. (**e**,**f**) The bar chart shows the metabolites that significantly increased and significantly decreased in the overweight group compared to the normal group; the *t*-test was used to evaluate the statistical differences between different conditions. * *p* < 0.05, ** *p* < 0.01 (Mann–Whitney-Wilcoxon test).

Genome-centric metabolic reconstruction was employed to compare the functional profiles of the gut microbiomes between the overweight and normal-weight groups. Metabolic pathway analysis links microbial community structure to functional potential, revealing key biological processes and metabolic activities associated with phenotypes, thereby clarifying the mechanisms by which gut microbiota influence host health. This analysis identified 54 gut metabolic pathways with significantly different abundances among the 377 species analyzed (*p* < 0.05; Table S4). These differential metabolic pathways were classified into 8 functional categories: amino acids, saccharides, energy, nitrogen, steroid hormones, short-chain fatty acids, nucleotides, and lipid metabolism. Notably, all 54 identified differential metabolic pathways were significantly down-regulated in the overweight group compared to the normal-weight controls (*p* < 0.05, Figure 3a,b). Key down-regulated pathways primarily involved amino acid biosynthesis and metabolism (e.g., ARGSYN-PWY, ARGSYNBSUB-PWY, ASPASN-PWY, COMPLETE-ARO-PWY, GLUTORN-PWY, PWY-2941, PWY-6629, PWY-7400) and fatty acid and lipid biosynthesis metabolism (e.g., PWY-5367, PWY-5667, PWY-5989, PWY-6284).

### 3.3. The Serum Metabolomic Profiles of Overweight Individuals

Non-metric multi-dimensional scaling (NMDS) analysis of the serum metabolome revealed a clear separation of profiles between the normal-weight and overweight cohorts, indicating a significant difference in their overall metabolic structures (stress < 0.2; Figure 3c). Metabolites with higher content in the overweight group than in the control group were defined as upregulated, whereas those with lower content in the overweight group were defined as downregulated. A total of 27 differential metabolites were identified (*p* < 0.05, VIP > 1), comprising 17 up-regulated and 10 down-regulated metabolites in the overweight group compared to the controls (Figure 3d & Table S5). These metabolites primarily belong to classes such as organic acids, their derivatives, and fatty acids. Specifically, the relative abundances of phenylalanylphenylalanine, L-alanine, L-leucine, pyrrolidine, propionylcarnitine, L-isoleucine, and picolinic acid were significantly elevated in overweight individuals (*p* < 0.05, Figure 3e). Conversely, the relative abundances of gentisaldehyde, perillic acid, beta-cryptoxanthin, citraconic acid, and 2-ethylpyrazine were significantly lower in the overweight group than in the normal-weight group (*p* < 0.05; Figure 3f).

### 3.4. Association Study Between Serum Metabolism and Gut Microecological Features in Overweight and Normal Groups

**Figure 4:**
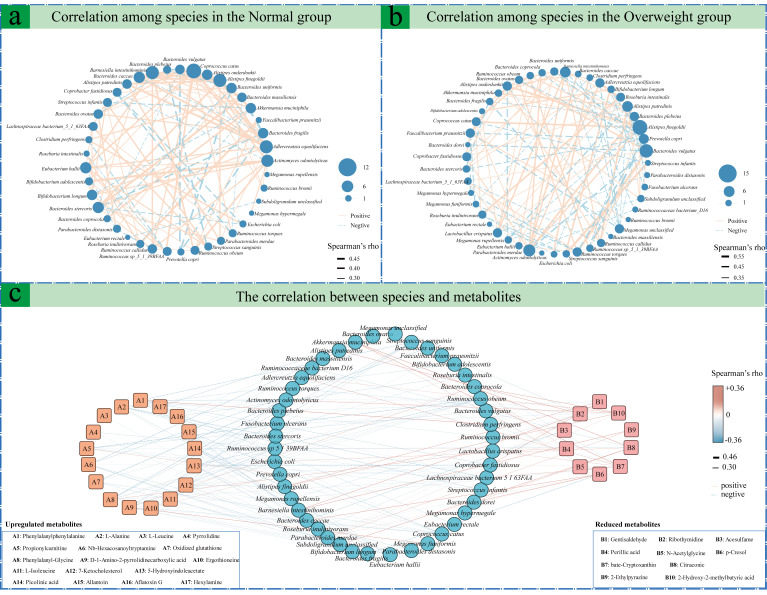
Correlation analysis of serum metabolites and gut microecological features between the overweight group and the normal group. (**a**,**b**) The correlation network diagrams respectively represent the correlations of the significantly different species in the gut of the overweight group and the normal group. (**c**) Correlation analysis between the significantly different species and the significantly different metabolites in the two groups. The squares on the left and right respectively represent metabolites with significant upregulation and significant downregulation, while the circle in the middle indicates the significantly different species in the intestine. The larger the circle, the higher the relative abundance of that species.

To elucidate the complex interactions between the gut microbiota and host metabolism. Spearman’s rank correlation analysis was performed between the relative abundances of microbial taxa and the contents of serum metabolites, as well as among microbial taxa, to identify significant intra-microbial relationships and associations between microbial taxa and differential serum metabolites in both the normal-weight and overweight groups (significance thresholds: *p* < 0.05, |r| ≥ 0.3). Within the normal-weight group, the analysis revealed several significant positive correlations (Figure 4a), including *Alistipes onderdonkii*, *Alistipes finegoldii*, *Adlercreutzia equolifaciens*, and *Barnesiella intestinihominis* (*p* < 0.05). *Bifidobacterium longum* was also positively correlated with *Bacteroides fragilis* and *Bifidobacterium adolescentis*. In contrast, *Parabacteroides distasonis* was significantly negatively correlated with *Eubacterium hallii* (*p* < 0.05). Distinct correlational patterns were observed in the overweight cohort (Figure 4b). *Lactobacillus crispatus* was positively correlated with *Bifidobacterium adolescentis* and *Alistipes finegoldii* but was significantly negatively correlated with *Parabacteroides distasonis* (*p* < 0.05). Furthermore, *Prevotella copri* demonstrated a significant positive correlation with *Bacteroides vulgatus* and *Bacteroides stercoris*, whereas *Bacteroides ovatus* was negatively correlated with these two species (*p* < 0.05). The analysis of microbe-metabolite interactions identified numerous significant associations. *Bifidobacterium longum* abundance was significantly negatively correlated with several metabolites, including L-leucine, pyrrolidine, phenylalanylphenylalanine, L-alanine, propionylcarnitine, and ergothioneine (*p* < 0.05). Conversely, *Escherichia coli* exhibited a significant positive correlation with metabolites phenylalanylphenylalanine, 5-hydroxyindoleacetate, and ergothioneine (*p* < 0.05). *Bifidobacterium adolescentis* was significantly negatively correlated with L-alanine, whereas *Bacteroides fragilis* was negatively correlated with beta-cryptoxanthin, p-cresol, and nb-hexacosanoyltryptamine (*p* < 0.05). Finally, *Akkermansia muciniphila* demonstrated a significant positive correlation with metabolites, citraconic acid and 2-hydroxy-2-methylbutyric acid (Figure 4c).

### 3.5. RFM Construction Evaluated the Contribution of Species, Pathways and Metabolites

**Figure 5:**
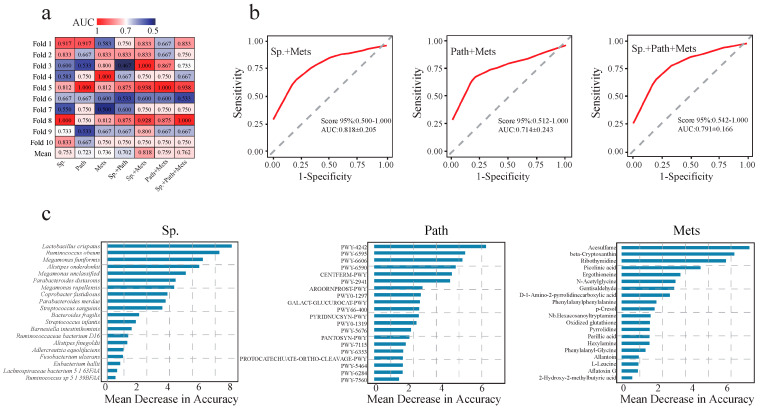
Significant differences in species and metabolites are associated with being overweight and possess diagnostic capabilities. (**a**) This heatmap presents the species, metabolic pathways and metabolites with significant differences among individual classifiers, as well as the 10-fold cross-validation feature curve (AUC) values of metabolites and the integrated classifier after combining individual classifiers. Sp., species, Path, metabolic pathways, Mets, serum metabolites. (**b**) Receiver operating characteristic curves showing the performance of random forest classifiers constructed with different combinations of metagenomic and metabolomic features in distinguishing overweight from controls in 10-fold cross-validation. The model combining metabolites, and species achieved the highest area under the curve (AUC = 0.833). (**c**) Feature importance analysis identifying the top twenty contributors from each data type.

To evaluate the diagnostic potential of different data types, a random forest model was constructed, and the performance of classifiers based on individual and combined features was quantified using the area under the receiver operating characteristic (ROC) curve (AUC). First, 10-fold cross-validation was performed on the training dataset to assess the generalizability of all classifiers (Figure 5a). For single-omics classifiers, the model based on microbial species composition achieved the highest predictive accuracy, with an average AUC of 0.753, followed by the serum metabolite-based classifier (average AUC = 0.736) and the gut microbial pathway-based classifier (average AUC = 0.723). For integrated multi-omics classifiers, the model combining species and metabolite data exhibited the best performance, with an average AUC of 0.818, and all integrated models achieved average AUC values exceeding 0.70 in cross-validation. Next, the three top-performing models from cross-validation were selected for independent validation on a held-out validation dataset (Figure 5b). The results showed that the species + metabolites classifier achieved an AUC of 0.818 in the independent validation set. In comparison, the pathway+ metabolites classifier yielded an AUC of 0.714, and the model integrating all three feature sets (species + pathway + metabolites) produced an AUC of 0.791. Notably, the AUC values in Figure 5a,b were derived from two distinct datasets, therefore minor numerical differences are expected. Critically, all models achieved AUC values > 0.70 in both analyses, which consistently underscores the strong capability of gut microbiota and serum metabolite profiles in discriminating between different body weight statuses. Additionally, the top 20 features with the highest predictive contributions were identified for each data type (Figure 5c). The key discriminatory species were *Ruminococcus obeum*, *Megamonas funiformis*, *Alistipes onderdonkii*, and *Parabacteroides distasonis.* The most influential metabolic pathways were PWY-4242, PWY-6595, PWY-6606, and CENTFERM-PWY. Among the metabolites, acesulfame, beta-cryptoxanthin, ribothymidine, picolinic acid, N-acetylglycine, and ergothioneine had the highest predictive contributions.

## 4. Discussion

The Mongolian population has long maintained a unique dietary culture characterized by high calorie intake, which was historically balanced by the high levels of physical activity inherent to nomadic pastoralism [28]. However, rapid urbanization and substantial lifestyle changes in recent decades have resulted in reduced physical activity and a dietary shift toward Westernized, energy-dense foods. Consequently, the prevalence of overweight and obesity has increased sharply in Mongolia. Furthermore, changes in body weight induce corresponding alterations in the gut microbiota [29]. In the present study, the random forest model demonstrated the highest predictive power when integrating microbial species and serum metabolite data. Subsequent identification of the most contributory features revealed that *Ruminococcus obeum*, *Alistipes onderdonkii*, and *Parabacteroides distasonis*, along with metabolites such as beta-cryptoxanthin, p-cresol and picolinic acid, are promising biomarkers for predicting overweight status. These findings provide a robust theoretical basis for the future development of diagnostic and risk prediction tools for overweight individuals.

Overweight and obesity are closely linked to metabolic disorders and altered gut microbiota composition. Although obesity has been extensively investigated in microbiota-related studies, research specifically focusing on overweight individuals remains limited. The observation of lower gut microbial diversity in overweight individuals than in their normal-weight counterparts is consistent with previous research findings [30,31,32], suggesting that reduced microbial diversity may be a common feature of overweight individuals across different Asian populations. Furthermore, beta diversity analysis revealed a significant divergence in the fecal microbiota structure between the two cohorts, indicating that overweight individuals exhibit gut dysbiosis. This study found that the relative abundance of the *Veillonellaceae* was significantly higher in the overweight group. Previous research has established that *Veillonellaceae* and *Prevotellaceae* are the primary producers of succinic acid [33]. In addition, a notable reduction in the abundance of several genera belonging to the Firmicutes, was observed in the overweight group. The lower abundance of these Firmicutes genera may contribute to the reduction in overall gut microbial biodiversity observed in overweight individuals, consistent with the results of the previous study [34]. Notably, the findings related to Asian populations are not completely consistent with previous reports. Studies focusing on Asian populations have also documented that overweight individuals exhibit lower gut microbial diversity and dysbiosis [19,35,36]. Higher levels of Veillonellaceae (propionate-producing bacteria) can trigger chronic low-grade inflammation and lipid metabolism disorders, thus promoting the development of obesity [37], which is consistent with the findings in the Mongolian population. Within adipose tissue, succinic acid exerts an anti-lipolysis effect by binding to the homologous receptor, succinic acid receptor 1 (Sncr1), leading to fat accumulation [38]. The present study found that the abundance of specific species, namely *Parabacteroides distasonis* and *Parabacteroides merdae*, was significantly higher in the normal-weight population. Both *Parabacteroides distasonis* and *Parabacteroides merdae* have been reported to possess probiotic potential and can alleviate obesity and related metabolic disorders [39]. The other key species identified in this study have also been implicated in other metabolic diseases. *Barnesiella intestinihominis* has been suggested to have therapeutic potential for neurological diseases, such as cerebral small vessel disease, by modulating gut metabolites, which can influence neuroinflammatory and neurodegenerative processes [40]. Similarly, *Alistipes onderdonkii* exhibits immunomodulatory and anti-inflammatory properties relevant to obesity, inflammatory bowel disease, and cancer, highlighting its potential as a next-generation probiotic [41,42]. Qin et al. [43] reported that *Alistipes onderdonkii* was significantly and negatively correlated with waist circumference in an Asian population. In contrast, some studies have reported that the abundance of *Megamonas*, particularly *Megamonas funiformis* and *Megamonas hypermegale*, is significantly increased in obese individuals and is positively correlated with waist circumference, body weight, and BMI. [44]. Consistent with this, the present study found that the abundance of *Eubacterium hallii* was higher in normal-weight individuals. Research on novel probiotics for obesity-related diseases has indicated that *E. hallii* can enhance insulin sensitivity and increase energy expenditure, further supporting its role as a beneficial commensal [45].

Moreover, the present study was conducted in a Mongolian population, whose unique lifestyle and dietary habits may contribute to the specific gut microbial composition observed. Traditional Mongolian diets are typically rich in high-fat animal products, fermented foods such as koumiss and yogurt, and complex dietary fibers, which are known to profoundly shape the structure and function of the gut microbiota. Notably, several key discriminatory taxa identified in this study are representative short-chain fatty acid (SCFA)-producing bacteria, including Veillonellaceae, *Parabacteroides distasonis*, *Parabacteroides merdae*, and *Eubacterium hallii*. Veillonellaceae is a major propionate-producing family that has been associated with inflammation and metabolic dysregulation in obesity [37]. *Parabacteroides distasonis*, *Parabacteroides merdae*, and *Eubacterium hallii* [46] are intestinal butyrate-producing bacteria. They maintain intestinal barrier integrity, attenuate chronic low-grade inflammation, improve insulin sensitivity, and regulate energy metabolism through butyrate production [47,48,49]. The higher abundance of these beneficial short-chain fatty acid-producing species in normal-weight Mongolian individuals suggests that they may exert a protective effect against excessive weight gain by producing short-chain fatty acids and regulating host metabolic homeostasis.

Serum metabolomics analyses have previously revealed that the metabolism of branched-chain and aromatic amino acids is dysregulated in overweight individuals, and the circulating levels of these metabolites are correlated with body weight. Indeed, amino acid metabolism disorders are increasingly associated with the pathophysiology of obesity [50,51,52]. Among the serum metabolites annotated in this study, beta-cryptoxanthin was significantly more abundant in the normal weight cohort. As a potent antioxidant, β-cryptoxanthin protects cells from free radical damage, inhibits inflammatory cytokines, prevents lipid peroxidation, and mitigates obesity-induced oxidative stress. Clinical studies corroborate these findings, demonstrating that a higher intake of beta-cryptoxanthin is associated with reduced BMI and body fat [53]. The present study also observed that the levels of L-leucine and L-alanine were elevated in conjunction with increased body weight. This is notable because L-alanine may inhibit lipogenesis by activating specific signaling pathways, potentially reducing fat accumulation and ameliorating obesity-related metabolic disorders [54]. The study found that overweight and obese individuals had significantly higher plasma L-leucine concentrations, and L-leucine levels decreased following weight loss intervention, which was positively associated with changes in BMI [55]. This study also found that the phenylalanylphenylalanine levels were significantly higher in the overweight group. This aligns with research in pediatric populations, where phenylalanine concentrations are significantly higher in overweight and obese children [56], suggesting that sustained alterations in amino acid metabolism are linked to excess body weight [57,58]. Furthermore, P-cresol, a degradation product of tyrosine and phenylalanine metabolism [59], has been shown to reduce the body weight of high-fat diet-induced obese (DIO) mice when administered intraperitoneally at a dose of 50 mg/kg every other day for four consecutive weeks [60], suggesting a complex regulatory role that may represent a potential therapeutic target [61]. Ribothymidine has also been implicated in metabolic dysregulation; its urinary levels are significantly elevated in diet-induced hyperlipidemic rats, linking it to hyperlipidemia. Given that obesity frequently involves metabolic abnormalities, ribothymidine may be a relevant biomarker for metabolic imbalance in overweight populations [62].

In addition, random forest was chosen mainly because it can effectively capture non-linear relationships between variables, is relatively robust to high-dimensional microbiome data, avoids overfitting to some extent, and allows model interpretation through feature importance scores. The results of the single classifier showed that species exhibited higher predictive performance (AUC = 0.753). This was because the complexity and diversity of the gut microbiota structure provided richer predictive features for the random forest model, and due to its stability, it could better reflect the long-term physiological status and environmental interactions of the host. In contrast, individual metabolites were more susceptible to short-term fluctuations such as lifestyle and sampling conditions. A classifier combining microbial species and serum metabolite data achieved high diagnostic accuracy (AUC = 0.818), confirming the potential of an integrated multi-omics approach for biomarker identification. This not only validates the association between gut microbiome alterations and overweight status but also indicates that such analyses hold considerable promise as an effective tool for early diagnosis.

This study has several limitations. The relatively small sample size (n = 96) and imbalanced gender ratio may limit the generalizability of the findings. As the study population was restricted to the Mongolian population with a unique high-calorie dietary pattern, the results may not be directly applicable to other ethnic groups or populations with different dietary habits. A lack of metadata regarding lifestyle factors and dietary habits represents another clear limitation. Although a high protein diet was proposed for the participants, detailed dietary habits were not collected, making it unclear whether the observed results were driven by overweight status or other confounding lifestyle factors. Information on lifestyle factors, such as dietary intake and physical activity, was not collected, which may represent potential confounders for the gut microbiota and metabolite profiles. The predictive model was not validated in an independent cohort, which should be considered when interpreting the results. Future multi-center studies with larger and more diverse populations, standardized lifestyle data collection, independent external validation, and more advanced machine learning strategies would be helpful to further verify and strengthen the present findings.

## 5. Conclusions

In summary, this study employed a multi-omics approach, integrating metagenomics and metabolomics, to elucidate significant changes in the gut microbiota and serum metabolites that distinguish normal-weight from overweight individuals. Notably, the decreased abundance of Firmicutes genera may contribute to the reduction in overall gut microbial biodiversity in overweight individuals. This study identified several key species, including *Parabacteroides distasonis*, *Barnesiella intestinihominis*, *Alistipes onderdonkii*, *Megamonas funiformis*, and *Megamonas hypermegale*, and specific amino acid metabolic pathways that were differentially abundant between the two groups. Additionally, distinct metabolic signatures including beta-cryptoxanthin, phenylalanylphenylalanine, p-cresol, and ribothymidine were identified. Crucially, the random forest model demonstrated robust diagnostic potential, achieving maximum predictive accuracy when the microbial species data were combined with metabolite profiles. This study on gut dysbiosis in individuals with overweight has revealed associations between specific gut microbiota and potentially informative serum metabolites, which may be useful for the assessment of overweight-related factors in this population. The developed model provides a foundational framework for future research on microbiome-regulated interventions, enhancing the potential for timely screening and clinical management of weight-related health conditions in humans. This study offers valuable insights for the research and diagnosis of overweight conditions, underscoring the diagnostic power of integrated multi-omics data. This study offers valuable insights for the research and diagnosis of incipient overweight conditions, underscoring the diagnostic power of integrated multi-omics data.

## Data Availability

The original data presented in the study are openly available in the Genome Sequence Archive (Genomics, Proteomics & Bioinformatics 2025) in National Genomics Data Center (Nucleic Acids Res 2025), China National Center for Bioinformation/Beijing Institute of Genomics, Chinese Academy of Sciences (GSA: CRA034508) that are publicly accessible at https://ngdc.cncb.ac.cn/gsa (accessed on 19 December 2025).

## Supplementary Materials

The following supporting information can be downloaded at: https://www.mdpi.com/article/10.3390/microorganisms14050946/s1. Table S1. Sample statistics; Table S2. Metagenomic Sequencing Data Output Before and After Quality Filtering; Table S3. The gut microbiota in the overweight group showed significant differences compared to the normal group.; Table S4. Significantly differential gut metabolic pathways between normal and overweight populations; Table S5. Significantly differential metabolites between normal and overweight populations.
